# Dynamic task–based rehabilitation and time to loss of toilet ambulation in a palliative care unit: a retrospective cohort study

**DOI:** 10.1007/s00520-026-11462-8

**Published:** 2026-11-07

**Authors:** Ryotaro Okamoto, Yoshiaki Okamoto, Susumu Jyoyama, Yuma Sonoda

**Affiliations:** 1 Department of Rehabilitation, Ashiya Municipal Hospital, Ashiya, Hyogo Japan; 2 Department of Rehabilitation Science, Kobe University Graduate School of Health Sciences, Kobe, Hyogo Japan; 3 Graduate School of Nursing Science, Tsuruga Nursing University, Tsuruga, Fukui Japan; 4 Department of Future Social Medicine, Kobe University Graduate School of Medicine, Kobe, Hyogo Japan

**Keywords:** Palliative care, Cancer rehabilitation, Ambulation, Toileting, Advanced cancer, Retrospective study

## Abstract

**Purpose:**

To determine whether dynamic-task rehabilitation, compared with bedside-only or no rehabilitation, is associated with longer time to loss of toilet ambulation among patients with advanced cancer in a palliative care unit (PCU).

**Methods:**

This retrospective study included patients with cancer admitted to a PCU who died during hospitalization (January 2020–March 2024). Eligible patients had walked to an in-room fixed toilet and stayed ≥ 14 days. Patients were classified as dynamic-task rehabilitation (Ex; ≥ 2 sessions), bedside rehabilitation (BS; ≤ 1 dynamic-task session), or no rehabilitation (NI). The primary outcome was time from PCU admission to loss of toilet ambulation. Kaplan–Meier and multivariable Cox analyses were used; Fine–Gray analyses treated death as a competing event.

**Results:**

A total of 205 patients were analyzed (Ex, n = 78; BS, n = 65; NI, n = 62). Median time to loss of toilet ambulation was 27 days in Ex, 20 days in BS, and 14 days in NI. In adjusted Cox models (NI reference), Ex was associated with a lower hazard of ambulation loss (HR, 0.462; 95% CI, 0.320–0.667), whereas BS was not (HR, 0.828; 95% CI, 0.559–1.226). Results were consistent in competing-risk analyses (Ex vs NI: sHR, 0.461; 95% CI, 0.328–0.647).

**Conclusion:**

Patients able to receive dynamic-task rehabilitation had a longer time to loss of toilet ambulation during PCU hospitalization. This association may reflect rehabilitation content, patient characteristics, and the clinical feasibility of dynamic-task rehabilitation. Prospective studies are needed to clarify causality.

## Introduction

Palliative care aims to improve quality of life by addressing physical, psychosocial, and spiritual suffering and supporting patients in living as actively as possible until death, with attention to individual needs and preferences [1]. In this context, hope can be sustained even when patients have realistic expectations about advanced illness [2, 3]. Patients with advanced cancer have diverse life and treatment goals, including quality of life, survival, and disease control [4].

Among these goals, maintaining mobility and the ability to use the toilet is an important wish [5, 6]. Ambulation and toileting are closely tied to privacy and dignity and may influence quality of life and spiritual well-being [5, 7]. In patients with advanced cancer, mobility declines markedly 2–3 weeks before death [8, 9], and only about one-fifth remain able to ambulate to the toilet 1 week before death [9]. Supporting patients’ wish to “keep walking as long as possible” is therefore clinically important.

In end-of-life cancer care, palliative rehabilitation aims not at functional restoration but “to maintain the best possible level of activity aligned with the wishes of patients and families, regardless of prognosis, and to enable the highest attainable quality of life during that time.” [10, 11] Cancer rehabilitation may relieve symptoms [12–14], reduce fatigue, increase physical activity, and improve quality of life in palliative care units (PCUs) [15]. However, studies have largely evaluated symptoms and psychological outcomes, leaving limited evidence on ambulation maintenance, particularly toilet ambulation. In PCUs, rehabilitation is tailored to patients’ conditions and preferences, ranging from dynamic tasks such as standing and walking practice (exercise-based therapy) to bedside rehabilitation, but whether intervention content is associated with the trajectory of ambulation remains unclear. Some patients receive no rehabilitation because they or their families do not wish to receive it. Comparing these groups may clarify the relationship between rehabilitation content and maintenance of toilet ambulation in palliative care.

This retrospective study examined whether rehabilitation exposure in a PCU was associated with time to loss of toilet ambulation. We hypothesized that rehabilitation incorporating dynamic tasks would be associated with a longer time to loss of toilet ambulation than bedside-only rehabilitation or no rehabilitation.

## Methods

### Study design and setting

This was a single-center retrospective cohort study using routinely collected medical records from the palliative care unit of Ashiya Municipal Hospital, Japan. The 24-bed PCU exclusively admits patients with cancer. All patient rooms are private and equipped with an in-room fixed toilet approximately 5 m from the bedside, although the exact distance varies among rooms. Rehabilitation was provided on weekdays by hospital-based physical and occupational therapists who also covered general wards; none were assigned exclusively to the PCU.

### Participants and eligibility criteria

We included patients with cancer admitted to the PCU who died during hospitalization between January 1, 2020, and March 31, 2024. Eligible patients had at least one documented episode of ambulation to the in-room fixed toilet. We excluded patients with no documented ambulation during admission, insufficient documentation of rehabilitation content or ambulation status, or a PCU stay < 14 days. Stays < 14 days were excluded because rehabilitation was typically provided 1–2 times per week, limiting opportunities to evaluate differences in content, particularly dynamic tasks.

### Exposure classification and rehabilitation content

Groups were classified from medical records according to rehabilitation received from PCU admission until death. Patients receiving ≥ 2 dynamic-task sessions were classified as Ex; those receiving bedside rehabilitation without dynamic tasks or only one dynamic-task session were classified as BS. The cutoff of ≥ 2 dynamic-task sessions was defined a priori based on clinical practice. The first session primarily assessed physical function, symptoms, and rehabilitation feasibility, often limiting task-specific training. Repeated sessions allowed reassessment of day-to-day clinical variation and adjustment of training load and the environment; thus, ≥ 2 sessions distinguished repeated from single dynamic-task exposure. This cutoff was used as an operational definition of repeated dynamic-task exposure and was not intended to represent a sufficient rehabilitation dose. Rehabilitation was broadly offered unless general condition precluded participation; all included patients had been offered it. Patients who did not receive rehabilitation because they or their families did not wish to receive it were classified into the NI group. Rehabilitation content could change during hospitalization as clinical status and symptoms evolved. Rehabilitation was initiated after referral by the attending physician. At each session, therapists tailored content to physical condition, symptom burden, rehabilitation goals, and patient/family preferences, sharing information with the multidisciplinary team as needed. Dynamic tasks were introduced only when clinically feasible and consistent with patient goals, whereas bedside rehabilitation was selected when standing or walking practice was inappropriate or infeasible. For Ex and BS, components were selected through shared decision-making and tailored to symptom burden. Rehabilitation was typically provided 1–2 times weekly for 20–40 min per session. Bedside rehabilitation mainly consisted of upper- and lower-extremity therapeutic exercises, massage, range-of-motion exercises, or combinations thereof. Total session count, cumulative rehabilitation time, and cumulative dynamic-task sessions were not systematically available in routine records. We also determined time from PCU admission to the first and second rehabilitation sessions in Ex and BS and to the first and second dynamic-task sessions in Ex.

### Outcomes and definitions

The primary outcome was time to loss of toilet ambulation. Time zero was PCU admission, and the event date was the day toilet ambulation finally ceased. Because ambulation was documented daily, the last confirmed day was the “last ambulation day,” with the following day as the event date. Patients who remained ambulatory until death were treated as event-free and censored on the date of death. Secondary outcomes were (1) days from the last ambulation day to death and (2) the proportion of patients ambulatory at 14, 7, and 3 days before death and 1 day before death. This interval was the difference between the last toilet-ambulation day and death; smaller values indicated ambulation closer to death. Toilet ambulation was walking from the bedside to the fixed toilet, regardless of assistive-device use or human assistance; canes, walkers, and handrails were permitted. Walking only a few steps after wheelchair transport did not qualify as toilet ambulation. For patients who transitioned to wheelchair-assisted toileting or a bedside commode, the last day they walked to the fixed toilet defined the last toilet-ambulation day. Human-assistance level did not define the outcome because it varied across days and times of day and was not documented consistently enough for retrospective classification. FAC was a covariate for baseline walking function and assistance requirements, not part of the outcome definition. Toilet ambulation status was determined from nursing, rehabilitation, and physician records; nursing records generally documented toileting method daily. Missing documentation on a given day was not considered ambulation loss; surrounding records and transitions in toileting method were reviewed to determine the last ambulation day. When same-day records differed, toilet ambulation was considered present if any record explicitly documented walking to the fixed toilet. A single investigator reviewed all records using the same predefined procedure and electronic record search; no independent second review or inter-rater reliability assessment was performed.

### Covariates

Baseline characteristics included age, sex, cancer type, body mass index (BMI), Palliative Prognostic Index (PPI) [16], Eastern Cooperative Oncology Group Performance Status (PS) [17], and Functional Ambulation Category (FAC) [18]. PPI incorporates Palliative Performance Scale, oral intake, edema, resting dyspnea, and delirium; higher scores indicate poorer prognosis. PS ranges from 0 (fully active) to 4 (completely disabled); higher scores indicate poorer function. FAC classifies walking by required human assistance from 0 (nonfunctional ambulation) to 5 (independent ambulation); higher scores indicate greater independence.

### Statistical analysis

All analyses were performed using R (The R Foundation for Statistical Computing, Vienna, Austria). Tests were two-sided (*α* = 0.05). Continuous variables were compared using the Kruskal–Wallis test with post hoc Dunn tests and Holm adjustment, and categorical variables using the chi-square test or Fisher’s exact test when expected cell counts were < 5. Standardized mean differences (SMDs) quantified the magnitude of between-group imbalances. For the primary outcome, Kaplan–Meier curves were compared using the overall log-rank test and Holm-adjusted pairwise comparisons. Restricted mean survival time (RMST), which does not rely on the proportional hazards assumption, was estimated using a truncation time of 30 days. The 30-day horizon was selected as a clinically interpretable period spanning the group median times to ambulation loss. Pairwise RMST differences were estimated with 95% confidence intervals and Holm-adjusted p-values. Multivariable adjustment used a Cox proportional hazards model with group (Ex/BS/NI) as the primary exposure. Age, sex, BMI, PPI, PS, FAC, and cancer type were prespecified confounders entered using forced entry. Multicollinearity was assessed using the generalized variance inflation factor (GVIF), with GVIF^(1/(2Df)) for variables with multiple degrees of freedom. The proportional hazards assumption was evaluated using Schoenfeld residuals for each model term and the global test. Suggested covariate violations were assessed in sensitivity Cox models stratified by those covariates. Because death precludes subsequent toilet ambulation loss, death before loss was treated as a competing event in analyses complementary to the Cox model. Cumulative incidence functions (CIFs) were compared using Gray’s test, and subdistribution hazard ratios (sHRs) were estimated using a Fine–Gray model with the same covariates as the Cox model. The Cox model estimates the hazard of toilet ambulation loss among event-free patients, whereas the Fine–Gray model accounts for death in cumulative incidence. For days from last ambulation to death, between-group differences were assessed using the Kruskal–Wallis test with post hoc Dunn tests and Holm adjustment. Covariate-adjusted medians and between-group differences were estimated by median (τ = 0.5) quantile regression; marginal estimates averaged fixed-group predictions while preserving each patient’s covariates. A sensitivity analysis excluded within-group outliers using the IQR rule. For ambulatory proportions at 14, 7, and 3 days before death and 1 day before death, logistic regression models included group and the prespecified confounders, and adjusted group-specific proportions were estimated by marginal standardization. Risk differences (RDs) and 95% confidence intervals were estimated using bootstrap resampling (1,000–2,000 iterations), with Holm adjustment for pairwise comparisons. No data were missing among the 205 analyzed patients; all analyses therefore used complete cases.

### Ethics and consent

This study was approved by the Institutional Review Board of Ashiya Municipal Hospital (approval no. 37). This non-interventional retrospective chart review was conducted in accordance with the Declaration of Helsinki and applicable national guidelines. Patient data were managed using anonymized study IDs, and the linkage key was stored by the principal investigator under restricted institutional access. Study information, including the scope of eligible patients, purpose of data use, and right to withdraw without disadvantage, was posted on the hospital website to provide an opportunity to opt out.

## Results

### Study cohort and baseline characteristics

**Fig. 1:**
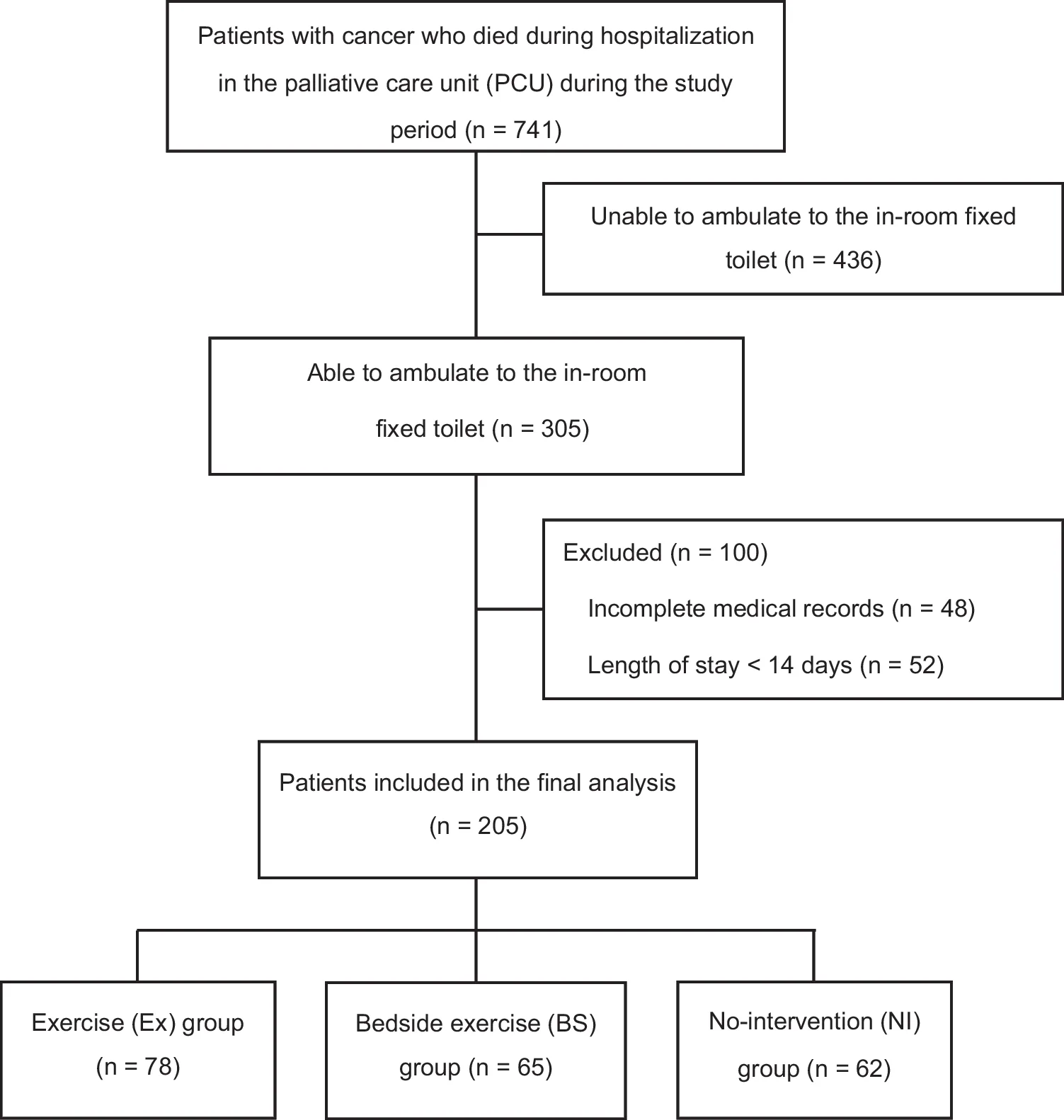
Flow of patient selection

A total of 205 patients were included in the final analysis (Ex, *n* = 78; BS, *n* = 65; NI, *n* = 62) (Figure 1).

Baseline characteristics are presented in Table 1. Standardized mean differences indicated small imbalances for age, BMI, and PPI but substantial imbalances for PS, FAC, and cancer type. These imbalances were addressed in the prespecified adjusted models.

**Table 1:** Baseline characteristics (*n* = 205)

Variable	Overall	Ex (*n* = 78)	BS (*n* = 65)	NI (*n* = 62)	SMD
Age, years, median [IQR]	79 [72.0, 87.0]	79 [71.2, 87.8]	78 [72.0, 85.0]	81 [75.0, 86.2]	0.094
Male sex, *n* (%)	88 (42.9)	30 (38.5)	28 (43.1)	30 (48.4)	0.134
BMI, kg/m^2^, median [IQR]	18.4 [16.6, 21.0]	18.1 [16.3, 21.1]	18.4 [17.0, 20.8]	18.8 [16.7, 21.6]	0.147
PS, *n* (%)					
1	29 (13.7)	14 (17.9)	8 (12.3)	7 (11.3)	0.410
2	26 (12.7)	11 (14.1)	10 (15.4)	5 (8.1)	
3	125 (61.3)	42 (53.8)	44 (67.7)	39 (62.9)	
4	24 (11.8)	11 (14.1)	3 (4.6)	10 (16.1)	
5	1 (0.5)	0 (0.0)	0 (0.0)	1 (1.6)	
FAC, *n* (%)					
0	36 (17.6)	16 (20.5)	6 (10.8)	14 (22.6)	0.479
1	11 (5.4)	3 (3.8)	4 (6.2)	4 (6.5)	
2	17 (8.3)	1 (1.3)	8 (12.3)	8 (12.9)	
3	58 (28.4)	24 (30.8)	19 (29.2)	15 (24.2)	
4	82 (40.2)	34 (43.6)	27 (41.5)	21 (33.9)	
PPI, *n* (%)					
< 4	99 (48.3)	39 (50.0)	35 (53.8)	25 (40.3)	0.238
4–6	64 (31.2)	25 (32.1)	20 (30.8)	19 (30.6)	
> 6	42 (20.5)	14 (17.9)	10 (15.4)	18 (29.0)	
Cancer type, *n* (%)					
Lungs	29 (14.1)	8 (10.3)	13 (20.0)	8 (12.9)	0.435
Stomach/esophagus	27 (13.2)	9 (11.5)	8 (12.3)	10 (16.1)	
Liver/gallbladder/pancreas	40 (19.5)	19 (24.4)	10 (15.4)	11 (17.7)	
Large intestine	41 (20.0)	12 (15.4)	16 (24.6)	13 (21.0)	
Breasts	9 (4.4)	5 (6.4)	1 (1.5)	3 (4.8)	
Kidneys/prostate/bladder	10 (4.9)	6 (7.7)	2 (3.1)	2 (3.2)	
Uterus/ovaries	17 (8.3)	7 (9.0)	7 (10.8)	3 (4.8)	
Other	32 (15.6)	12 (15.4)	8 (12.3)	12 (19.4)	

Continuous variables are presented as median [IQR], and categorical variables as *n* (%). SMD, standardized mean difference. Ex, dynamic-task rehabilitation; BS, bedside rehabilitation with ≤ 1 dynamic-task session; NI, no rehabilitation. p-values are intentionally omitted because the table is descriptive; imbalances were accounted for in adjusted models. Variables assessed at admission include age, sex, body mass index (BMI), Performance Status (PS), Functional Ambulation Category (FAC), Palliative Prognostic Index (PPI), and cancer type

### Timing of rehabilitation exposure

The median time from PCU admission to the first rehabilitation session was 3 days (Q1–Q3, 1–7) in the Ex group and 4 days (Q1–Q3, 1–6) in the BS group. In the Ex group, the median time from PCU admission to the first and second dynamic-task sessions was 4 days (Q1–Q3, 1–7.5) and 8 days (Q1–Q3, 6.3–12), respectively. respectively. The median time to the second rehabilitation session, irrespective of rehabilitation content, was 8 days (Q1–Q3, 6–12) in the BS group.

### Primary outcome: time to loss of toilet ambulation

**Fig. 2:**
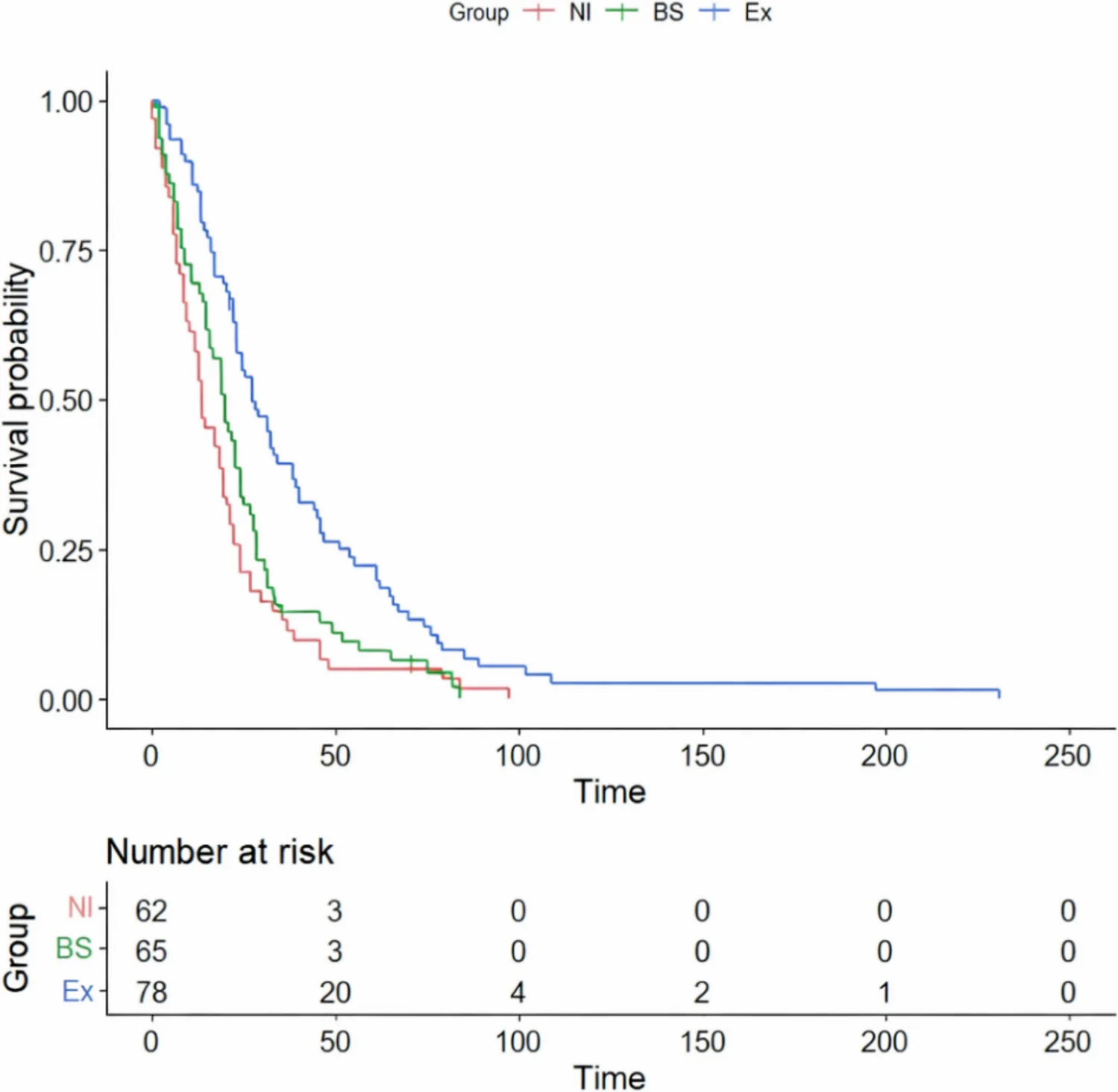
Kaplan–Meier curves for time to loss of toilet ambulation by group (Ex, BS, NI). The numbers at risk are shown below. Log-rank *p* < 0.001

Kaplan–Meier curves for time to loss of toilet ambulation by group are presented in Fig. 2.

Median times to loss of toilet ambulation (95% confidence intervals) were 27 (23–38) days in Ex, 20 (16–24) days in BS, and 14 (12–20) days in NI. The overall log-rank test was significant; pairwise comparisons were significant for Ex vs BS (*p* = 0.004) and Ex vs NI (*p* < 0.001), but not BS vs NI (*p* = 0.251). At 30 days, the RMST was 23.0 days (95% CI, 21.1–24.9) in Ex, 18.5 days (95% CI, 16.1–21.0) in BS, and 15.5 days (95% CI, 13.1–18.0) in NI. The RMST difference was 7.4 days (95% CI, 4.3–10.5; Holm-adjusted *p* < 0.001) for Ex − NI, 4.4 days (95% CI, 1.4–7.5; Holm-adjusted *p* = 0.010) for Ex − BS, and 3.0 days (95% CI, − 0.5 to 6.4; Holm-adjusted *p* = 0.090) for BS − NI.

### Adjusted analyses: Cox proportional hazards and sensitivity analysis

In the covariate-adjusted Cox model with NI as reference, the hazard ratio (HR) was 0.462 (95% CI, 0.320–0.667; *p* < 0.001) for Ex and 0.828 (95% CI, 0.559–1.226; *p* = 0.346) for BS. With BS as reference, Ex was associated with a lower hazard of toilet-ambulation loss (HR, 0.558; 95% CI, 0.377–0.826; *p* = 0.004). The maximum GVIF^(1/(2Df)) was 1.221, indicating no substantial multicollinearity. Schoenfeld residual testing indicated no proportional hazards violation for group (*p* = 0.246), and the global test was not statistically significant (*p* = 0.057). A violation was suggested for FAC (*p* = 0.005); therefore, a sensitivity Cox model stratified by FAC was performed. The HR for Ex vs NI was 0.447 (95% CI, 0.299–0.669), consistent with the primary analysis.

### Competing-risk analyses

**Fig. 3:**
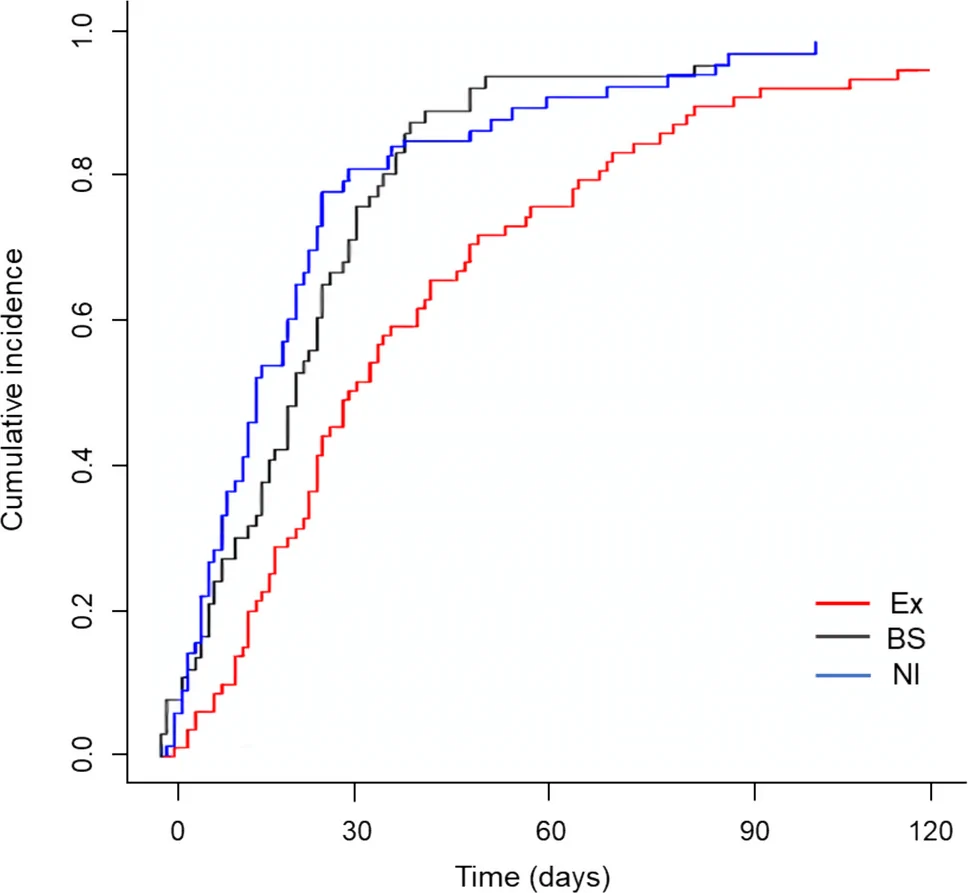
Cumulative incidence functions for loss of toilet ambulation by group, with death treated as a competing event

In competing-risk analyses, death before toilet ambulation loss was treated as the competing event. Cumulative incidence functions by group are shown in Fig. 3.

Gray’s test indicated a significant between-group difference (*p* = 0.003). In Fine–Gray regression, Ex vs NI remained significant (sHR, 0.461; 95% CI, 0.328–0.647; *p* < 0.001), as did Ex vs BS (sHR, 0.553; 95% CI, 0.385–0.794; *p* = 0.001), whereas BS vs NI was not significant (sHR, 0.834; *p* = 0.370).

### Secondary outcome: days from last toilet ambulation to death

Days from the last toilet ambulation day to death differed among groups (Kruskal–Wallis χ^2^ = 10.66, *p* = 0.005). Post hoc Dunn tests with Holm adjustment showed differences for BS vs Ex (*p* = 0.002) and Ex vs NI (*p* = 0.047), but not BS vs NI. In median (τ = 0.5) quantile regression with marginal standardization, the adjusted medians (95% CIs) were 8.5 (5.7–12.7) days in Ex, 14.2 (9.6–19.4) days in BS, and 10.5 (7.8–14.1) days in NI. Adjusted between-group median differences were − 5.5 (− 10.7 to − 0.2) days for Ex − BS, − 1.9 (− 6.1 to 2.7) days for Ex − NI, and + 3.7 (− 2.2 to 9.1) days for BS − NI. Sensitivity analyses excluding within-group outliers using the IQR rule yielded similar findings.

### Secondary outcome: ambulatory proportion at prespecified time points before death

**Fig. 4:**
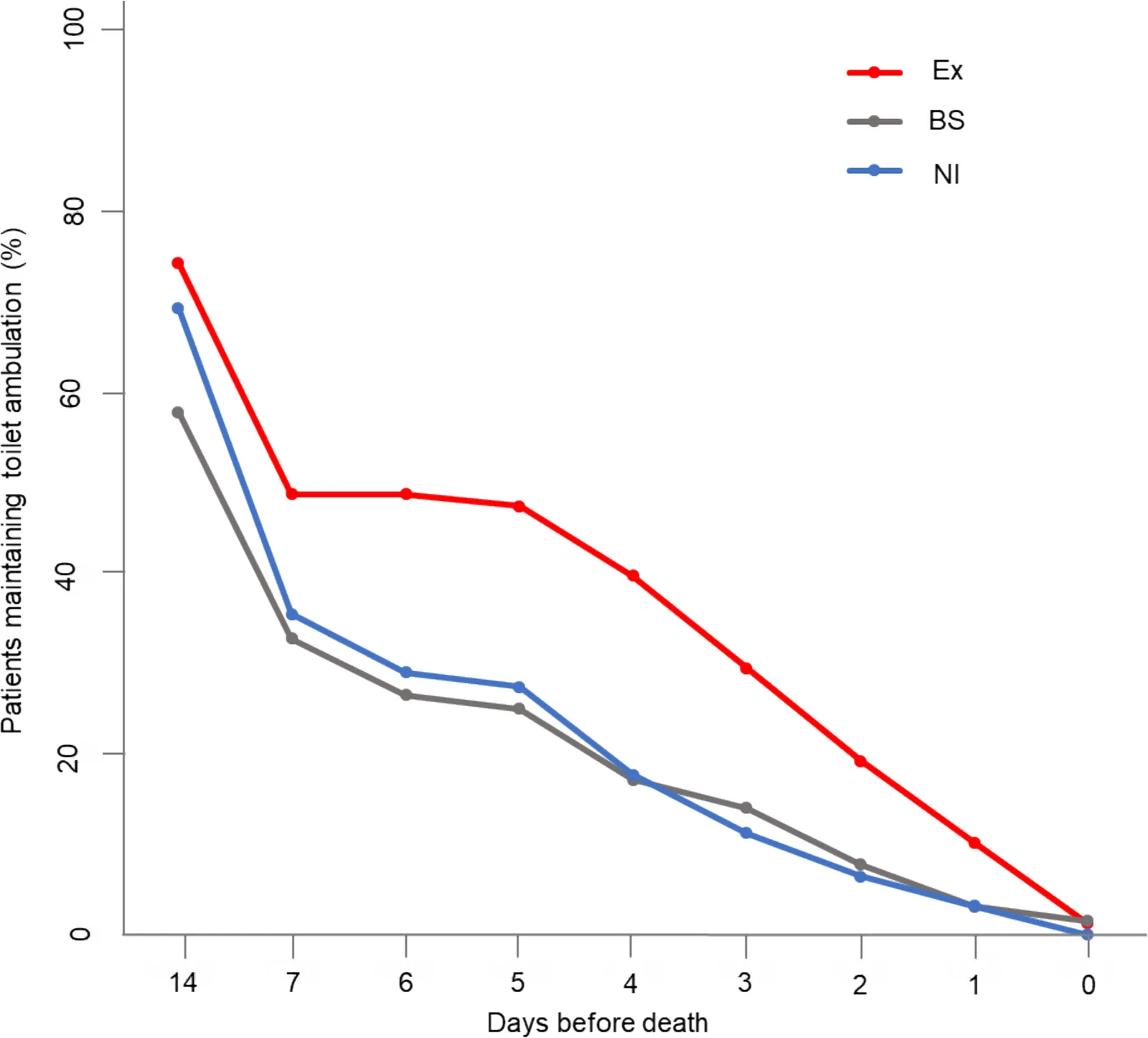
Adjusted proportions of patients maintaining toilet ambulation at each time point before death by group

Adjusted proportions of patients maintaining toilet ambulation at each time point before death are shown in Fig. 4.

Marginal standardized logistic regression with bootstrap resampling estimated adjusted ambulatory proportions and risk differences (RDs) at 14, 7, and 3 days before death and 1 day before death. At 14 days, the adjusted ambulatory proportion (95% CI) was 74.7% (64.5%–84.6%) in Ex, 56.6% (43.9%–69.4%) in BS, and 69.8% (57.8%–81.0%) in NI; the RD for Ex − BS was + 18.1% (+ 2.4% to +34.6%). At 7 days, adjusted proportions were 47.1% (35.4%–59.4%) in Ex, 33.4% (21.2%–46.3%) in BS, and 36.8% (24.9%–48.9%) in NI, with borderline or nonsignificant RDs. At 3days, adjusted proportions were 28.6% (18.4%–39.9%) in Ex, 14.7% (6.0%–24.8%) in BS, and 11.4% (3.4%–21.0%) in NI; the RD was + 17.2% (+ 3.1% to +31.4%) for Ex − NI and + 14.0% (− 2.2% to +29.0%) for Ex − BS. At 1 day, events were rare and estimates unstable.

## Discussion

### Principal findings

Patients who received rehabilitation incorporating dynamic tasks had a longer time to loss of toilet ambulation than those who received bedside rehabilitation without dynamic tasks or no rehabilitation. This association may reflect rehabilitation content and differences in patient condition, symptom burden, prognosis, and the clinical feasibility of dynamic-task rehabilitation. Confounding by indication, residual confounding from unmeasured clinical factors, and potential immortal time bias from classification by rehabilitation received during hospitalization also limit interpretation. No clear difference was observed between the BS and NI groups. Secondary outcomes were not fully consistent with the primary analysis. Although Ex had a higher adjusted ambulatory proportion at 3 days before death, this pattern was not consistent across time points; at 14 days, the adjusted proportion was higher in NI than BS. The adjusted interval from last toilet ambulation to death was shorter in Ex than in BS, whereas the Ex–NI difference was not statistically significant. These findings should therefore be interpreted cautiously and do not support a consistent ordering of Ex, BS, and NI throughout the period before death. Maintaining toilet ambulation may have patient-centred implications near the end of life [8, 9], but dignity, hope, and related outcomes were not directly assessed.

### Comparison with previous studies

Our findings are directionally consistent with reports of favorable physical and functional outcomes from exercise interventions in advanced cancer. Oldervoll et al. reported improved muscle strength, quality of life, and walking distance with twice-weekly exercise in patients with cancer and a prognosis < 6 months [19]. Nadler et al. [21] found that exercise improved physical function in advanced cancer with favorable safety profiles. Reviews by Toohey [13] and Tanriverdi [14] also support the feasibility and potential benefits of exercise in palliative care. The longer time to toilet ambulation loss in Ex is directionally consistent with these studies; however, the observational design does not permit causal effects of dynamic-task rehabilitation to be determined.

### Interpretation and clinical implications

No clear difference in time to toilet ambulation loss was observed between BS and NI, but this does not show that bedside-focused rehabilitation is insufficient. BS patients may have had greater symptom burden or poorer dynamic-task feasibility, which could also be associated with toilet ambulation loss. Frikkel et al. [20] identified fatigue, pain, and psychological barriers as factors limiting physical activity in advanced cancer, supporting consideration of patient condition and intervention feasibility when interpreting group differences. Cheville et al. [11] reported that comprehensive rehabilitation in advanced cancer was associated with prolonged maintenance of activities of daily living, underscoring the importance of appropriately targeted interventions. Although Ex patients had longer time to toilet ambulation loss than BS patients, this difference should not be attributed to dynamic tasks alone because patient condition and dynamic-task feasibility may also have contributed.

### Relevance to dignity and person-centered goals

Dependency and loss of autonomy can threaten dignity in advanced illness [5]. Maintaining toilet ambulation near the end of life may therefore have implications for dignity, hope, and psychological well-being. These outcomes were not directly assessed, and their association with toilet ambulation maintenance requires further study. Trials and meta-analyses suggest exercise may improve physical function in advanced cancer, although findings are not uniformly significant [19, 21].

## Limitations

First, the single-center retrospective design may entail selection bias, information bias, and regional practice effects. Second, rehabilitation content varied with symptom burden and patient preferences, and dynamic-task feasibility depended on general condition and symptom severity. Confounding by indication cannot be excluded because patients in better condition may have been more likely to enter Ex. Third, classification by rehabilitation received over the entire hospitalization required patients to survive and remain able to receive ≥ 2 dynamic-task sessions before entering Ex. The second dynamic-task session occurred at a median of 8 days (Q1–Q3, 6.3–12) after PCU admission. Patients with early toilet-ambulation loss were therefore less likely to enter Ex, potentially introducing immortal time bias and overestimating the observed association. Because we did not perform a time-dependent exposure analysis or landmark analysis, this potential immortal time bias could not be eliminated. Despite adjustment for major prognostic and functional variables and consistent findings across analytic approaches, including competing-risk models, residual confounding by unmeasured clinical factors cannot be excluded. Pain, fatigue, dyspnea, delirium or impaired consciousness, nutrition, oxygen use, medications (including opioids and sedation), psychological factors, and patient/family preferences may have influenced both dynamic-task feasibility and toilet-ambulation maintenance. Finally, although frequency and session duration followed the institutional protocol, total sessions, cumulative rehabilitation time, and cumulative dynamic-task sessions were not systematically recorded. We therefore could not determine whether between-group differences reflected rehabilitation content, dose, or both. Future prospective or multicenter studies should quantify intervention dose, symptom trajectories, and safety and include patient-centered outcomes such as quality of life and dignity.

## Conclusions

Patients able to receive dynamic-task rehabilitation had a longer time to loss of toilet ambulation during PCU hospitalization. However, patient characteristics and the clinical feasibility of dynamic-task rehabilitation may have contributed to this observed association, and causal effects cannot be determined from the present retrospective study.

## Data Availability

The datasets analyzed during the current study are available from the corresponding author on reasonable request.
